# Prediction Model for the Risk of HIV Infection among MSM in China: Validation and Stability

**DOI:** 10.3390/ijerph19021010

**Published:** 2022-01-17

**Authors:** Yinqiao Dong, Shangbin Liu, Danni Xia, Chen Xu, Xiaoyue Yu, Hui Chen, Rongxi Wang, Yujie Liu, Jingwen Dong, Fan Hu, Yong Cai, Ying Wang

**Affiliations:** 1 School of Public Health, Shanghai Jiao Tong University School of Medicine, Shanghai 200025, China; dyq1997@hotmail.com (Y.D.); liushangbin@sjtu.edu.cn (S.L.); dannix0101@163.com (D.X.); xuchen233333@163.com (C.X.); dd2192003@163.com (X.Y.); chenhui608@sjtu.edu.cn (H.C.); RosieW816@outlook.com (R.W.); liuyj_4287@163.com (Y.L.); ilovemath@sjtu.edu.cn (J.D.); 2 Department of Occupational Health, School of Public Health, China Medical University, Shenyang 110122, China

**Keywords:** men who have sex with men, nomogram, machine learning, HIV infection, model validation, psychosocial factors, involuntary subordination

## Abstract

The impact of psychosocial factors on increasing the risk of HIV infection among men who have sex with men (MSM) has attracted increasing attention. We aimed to develop and validate an integrated prediction model, especially incorporating emerging psychosocial variables, for predicting the risk of HIV infection among MSM. We surveyed and collected sociodemographic, psychosocial, and behavioral information from 547 MSM in China. The participants were split into a training set and a testing set in a 3:1 theoretical ratio. The prediction model was constructed by introducing the important variables selected with the least absolute shrinkage and selection operator (LASSO) regression, applying multivariate logistic regression, and visually assessing the risk of HIV infection through the nomogram. Receiver operating characteristic curves (ROC), Kolmogorov–Smirnov test, calibration plots, Hosmer–Lemeshow test and population stability index (PSI) were performed to test validity and stability of the model. Four of the 15 selected variables—unprotected anal intercourse, multiple sexual partners, involuntary subordination and drug use before sex—were included in the prediction model. The results indicated that the comprehensive prediction model we developed had relatively good predictive performance and stability in identifying MSM at high-risk for HIV infection, thus providing targeted interventions for high-risk MSM.

## 1. Introduction

In 2020, there are 38 million people living with HIV worldwide, thus making HIV infection one of the most serious public health problems worldwide. Men who have sex with men (MSM) account for an integral part of new HIV infections, which has posed an unprecedented challenge to global HIV prevention and control [1]. In China, MSM also constitute a key population at high risk of HIV infection. The risk factors of HIV infection in MSM identified by current research mainly include sociodemographic, behavioral, and psychosocial aspects, such as sex, age, unprotected anal intercourse (UAI), multiple sexual partners (MSP), alcohol and drug use before sex and depression [2,3]. Given the variety of risk factors of HIV infection among MSM, identifying MSM at greatest risk for HIV infection by using predictive models could facilitate targeted prevention interventions specific to this population, while permitting more focused delivery of prevention resources. Therefore, it is necessary to predict the risk of HIV infection in a more holistic perspective for early targeted screening and prevention among MSM.

Since sexual behavior is considered as the main way of HIV transmission and infection among MSM, most HIV prevention interventions have focused on directly reducing unprotected sexual behavior, while neglecting the impact of psychological factors related to HIV infection. As a sexual minority group, the MSM is subject to stigma, discrimination, and isolation from society, which in turn causes a range of defensive social mentality encompassing cognition, feelings and behaviors, and ultimately contributes to various psychosocial problems [4,5]. The resulting psychosocial problems may lead to high-risk behaviors-such as UAI and MSP, which might provide immediately physical or emotional gratification but could increase the risk of HIV transmission and infection in the long run.

Considering the characteristics of MSM and the surrounding social environment, involuntary subordination, sexual impulsivity and social support are emerging as new public health concerns among MSM [6,7,8]. Involuntary subordination (IS) is a mechanism adapted by humans for competing resources that switch off fighting behaviors when a losing organism cannot struggle anymore (thus saving the organism from injury) [9]. Individuals with high levels of IS are more difficult to accept defeat or escape from a defeated situation, and then give up resistance or abandon themselves. This possibly makes MSM vulnerable to HIV infection. However, there are few studies focused on direct relationship between involuntary subordination and HIV infection among MSM. Recently, a cross-section study revealed that high level of IS was associated with multiple sexual partners [10]. As we all know, multiple sexual partners, as a sexual risk behavior, may increase the risk of HIV infection. Therefore, we speculate that IS, as a psychosocial variable associated with sexual behavior, may be a significant predictor of HIV infection among MSM. Sexual compulsivity (SC) is a trait characterized by sexual fantasies and behaviors that increase in intensity and frequency over time and, therefore, interfere with personal, interpersonal, or career pursuits [11,12]. Compared with MSM lacking this characteristic, MSM with sexual impulsivity experienced a higher incidence of HIV and sexually transmitted infections [13]. The MSM engaging in compulsive sexual behavior may also partly stem from the pressures and stigma they feel as a sexual minority. In addition, some evidence suggested that high levels of social support were associated with decreased likelihood of engaging in sexual risk behaviors [14,15,16,17]. Therefore, social support that can provide emotional and material resources to MSM could help reduce the risk of HIV infection [18,19]. In summary, especially among MSM, multidimensional factors, particularly emerging psychosocial factors, need to be considered in predicting the risk of HIV infection.

There are many studies at home and abroad that have developed HIV infection risk assessment tools targeting MSM, including the Menza score [20], the Denver score [21], and the San Diego Early Test score [22]. In addition, prediction models specific to Chinese MSM have been developed to estimate HIV infection risk [23,24]. However, all the models mentioned above included only socio-demographic and behavioral factors, not psychosocial factors, thus could not comprehensively predict the risk of HIV infection among MSM. To our knowledge, there are few studies using machine learning methods to construct multidimensional HIV infection prediction models that include emerging psychosocial variables. In this study, we aimed to establish and validate an integrated HIV infection risk assessment tool that incorporated variables selected from demographic, psychosocial and behavioral factors using machine learning approach, and inform targeted interventions in MSM.

## 2. Methods

### 2.1. Study Population and Eligibility Criteria

**Figure 1:**
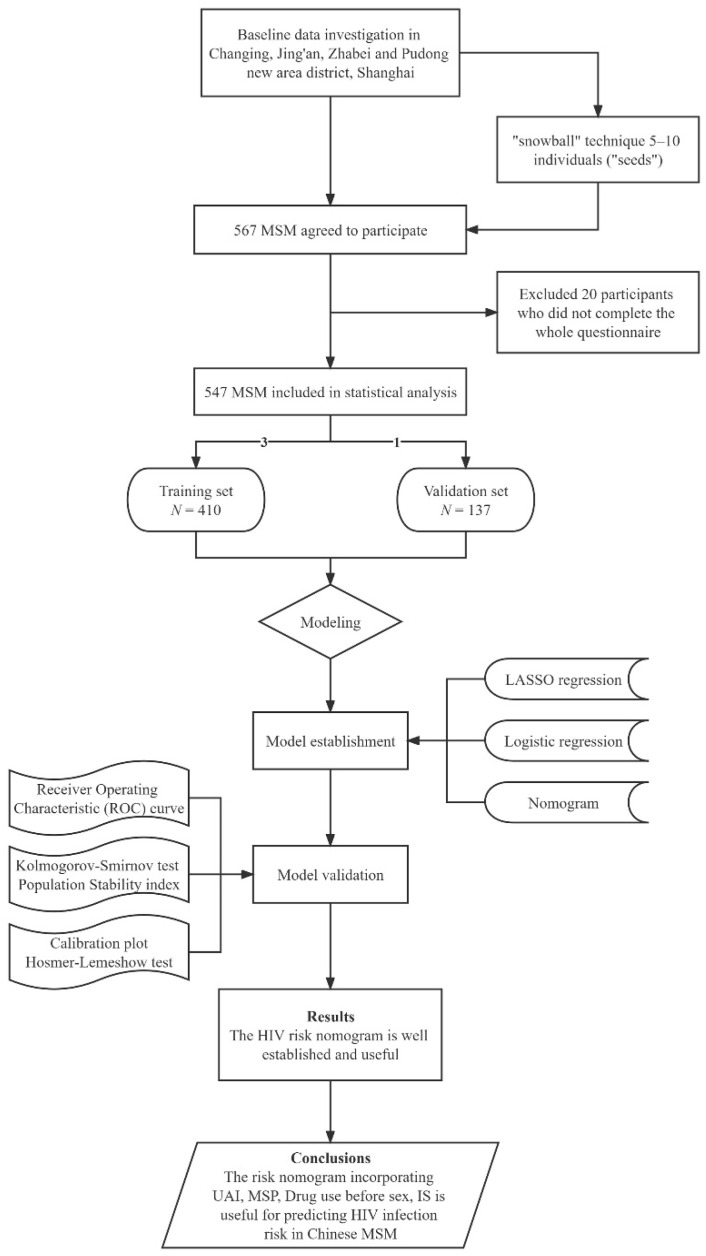
Flow diagram of study design.

A cross-sectional study was performed among MSM in four districts: Changning, Jing’an, Zhabei and Pudong New Area in Shanghai. Individuals were eligible to participate only if they were biologically male, at least 18 years of age, and reported to have had sex with men during the past 6 months. A total of 567 MSM agreed to participate in this study, and 20 MSM were not included in the final statistical analysis due to not completing the whole questionnaire. The final participants for assessment thus included 547 MSM (response rate of 96%). A flow diagram of the study design is shown in Figure 1.

### 2.2. Participants and Procedure

The “snowball” technique, which is advantageous in addressing population covertness, was adopted to target the participants. Initially, with the help of the local Center for Disease Control and Prevention and non-government organizations, we targeted 5 to 10 individuals (“seeds”) compatible with the inclusion criteria within each district. Subsequently, those harder-to-reach MSM of the same social-cultural background were identified through the indication of the so-called “seeds”, and so forth, forming the snowball. Anonymous face-to-face interviews were carried out between our outreach workers and the participating MSM. Participants were briefed about the study in detail. Written informed consent was obtained from the participants before the interview. Interviewers were requested to sign a form pledging that they had made clear explanations to the participants and answered all questions before the participants signed the informed consent. We made sure that, in the whole process of dealing with our participants, we strictly complied with American Psychological Association ethical standards. The recruitment procedure and the design of this study were approved by the Ethics Committee of School of Public Health, Shanghai Jiao Tong University.

### 2.3. Measurement

#### 2.3.1. Socio-Demographic Variables

Socio-demographic information collected included age, income, current marital status, employment status, education level, registered residence status, self-reported sexual orientation.

#### 2.3.2. Psychosocial Variables

##### Involuntary Subordination

Involuntary subordination was measured using the 32-item, 5-point Likert-type Involuntary Subordination Questionnaire [9]. Answers were rated ranging from 1 (strongly disagree) to 5 (strongly agree) where a higher total score indicates a higher level of involuntary subordination (Cronbach’s alpha coefficient = 0.883; range 35–136).

##### Sexual Compulsivity

The 10-item Likert-type Sexual Compulsivity Scale was applied to measure individual’s out-of–control sexual thoughts and behaviors [25]. Answers were rated ranging from 1 (strongly disagree) to 4 (strongly agree), with a higher total score indicating higher sexual compulsivity (Cronbach’s alpha coefficient = 0.854; range 10–40).

##### Social Support

We employed the Multiple Scales of Perceived Social Support to assess subjectively recognized social support from family, friends, and significant others [26,27]. The scale is a 7-point Likert scale with 12 items, ranging from 1 (very strongly disagree) to 7 (very strongly agree). A higher total score indicated higher social support (Cronbach’s alpha coefficient = 0.932; range 18–84).

#### 2.3.3. Behavioral Variables

##### Multiple Sexual Partners

We asked participants “How many male sexual partners have you had anal sex with in the past 6 months?”. The options included “only one”, “two”, “three”, “four”, and “five or more”. We classified those who answer this question with more than one male partner as having existing MSP.

##### Unprotected Anal Intercourse

Participants were asked about the frequency of condoms use when they had sex with any male partner in the past 6 months. Responses were rated ranging from 1 (never) to 5 (every time). Unless the answer was “every time”, they were described as “engaging in UAI”.

##### Alcohol Use before Sex

We measured drug use by asking the participants whether they had drinks before sex. Those without a “never” answer was regarded as groups who drink before sex.

##### Drug use before Sex

We measured drug use by asking the participants whether they had used any drugs including ecstasy (3,4 methylenedioxymethamphetamine), crystal meth (methamphetamine), marijuana, cocaine, and others. Those without a “never” answer was regarded as groups who use drugs before sex.

##### Voluntary HIV Counseling and Testing (VCT)

Participants were asked if they had voluntarily received HIV counseling and testing in the past six months, and participant’s blood samples were voluntarily drawn and tested for HIV infection. Then, they were privately informed of their serostatus. If participants have a positive result, they are referred to a local hospital specializing in HIV/AIDS treatment.

### 2.4. Statistical Analysis

The 547 participants were randomly divided into a training set and a testing set for internal validation at the theoretical ratio of 3:1 [28,29]. The data from the training and testing sets were guaranteed not to be replaced during the analysis, thus improving the reliability and robustness of the study findings. Categorical variables are presented as frequencies and percentages and continuous variables are described as mean and SD (normal distribution) or median and quartile (non-normal distribution). Chi-square test, corrected Chi-square test, and Fisher exact test were used to assess differences in categorical variables between training and testing set. Differences in continuous variables between the training and testing set were evaluated using Unpaired t test (normal distribution) and Mann–Whitney U test (non-normal distribution). Previous studies have shown that least absolute shrinkage and selection operator (LASSO) regression analysis outperformed several other machine learning approaches [30,31,32]. Therefore, LASSO was adopted to reduce the data dimension of the training set and to pick out the most influential and optimal factors for the predictive model. LASSO regression analysis minimizes the prediction error of continuous dependent variables by imposing constraints on the model parameters that causes the regression coefficients of certain predictor variables to shrink to zero. Variables with non-zero regression coefficients are considered risk factors that are strongly correlated with the response variable. On the contrary, predictor variables with regression coefficients equal to zero after the contraction process are excluded from the model. The 10-fold cross-validation solves the problem of overfitting variables and multicollinearity during model extrapolation by centralizing and standardizing the included variables for optimizing lambda. After the risk factors were identified by LASSO regression analysis, a prediction model was constructed by using multiple logistic regression analysis on the training set, which was presented in the form of a nomogram. Then, a predictive nomogram based on identified risk factors was established as a tool to assess HIV infection risk visually and quantitatively among MSM.

The discrimination, calibration and stability of the HIV infection risk nomogram were thoroughly assessed based on the training set and the testing set, respectively. Discrimination of nomogram was quantified using the area under the curve (AUC) of the receiver operating characteristic (ROC) and Kolmogorov–Smirnov (K-S) test. The Kolmogorov–Smirnov test was used to assess the agreement between the predicted and actual probabilities of HIV infection risk and higher K-S values indicating greater ability of the model to discriminate the samples. It is generally considered that K-S > 0.2 denotes a strong risk differentiation ability of the model developed in the study. The calibration plots and Hosmer–Lemeshow test were used to evaluate the calibration of prediction model. Internal and external consistency of the discrimination and calibration performance were measured using bootstrap resampling procedure. In addition, population stability index (PSI) was introduced to monitor model stability by assessing the degree of population bias based on the difference between the expected distribution of the training sample and the actual distribution of the test sample. Lower PSI means smaller difference between the two datasets and better stability of the model, and generally considered as PSI between 0 and 0.1, indicating no significant population bias and excellent stability of the model.

All analyses were conducted with R statistical software (version 3.6.1) equipped with the “caret”, “glmnet”, “rms”, “rmda” packages. All tests were two-sided and *p* ≤ 0.05 was set as the level of significant difference.

## 3. Results

### 3.1. Characteristics of Participants

Among the 547 MSM in our study, 59 were diagnosed as HIV-positive (including 24 previously HIV-positive participants). The average age of the participants was 28.0 (25.0–33.0) years old. The specific demographic, psychosocial and behavioral characteristics in the training set and testing set were summarized in Table 1. There is no statistically significant difference between the characteristics of the training and testing datasets, which indicates that the random assignment to each subset is successful.

### 3.2. Construction and Development of a Risk Assessment Tool for HIV Infection among MSM

**Figure 2:**
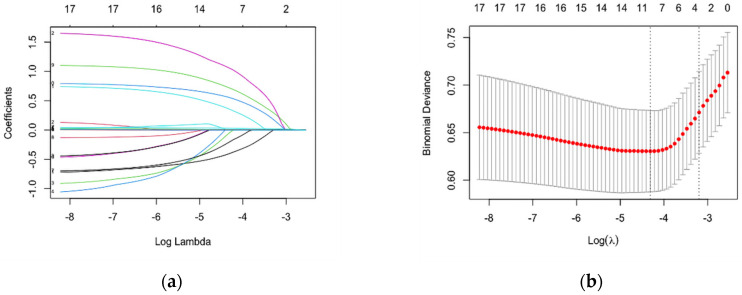
Predictors selection using the least absolute shrinkage and selection operator (LASSO) binary logistic regression model: (**a**) A coefficient profile plot was constructed against the log (lambda) parameters. Four variables with nonzero coefficients were selected by deriving the optimal lambda; (**b**) Selection of optimal parameter (lambda) in the LASSO model used 10-fold cross-validation error curve and was based on 1 standard error of the minimum criteria (1-SE criteria). The partial likelihood deviance (binomial deviance) curve was plotted versus log (lambda). The dotted lines were drawn at the optimal values by the minimum criteria and 1-SE criteria.

**Figure 3:**
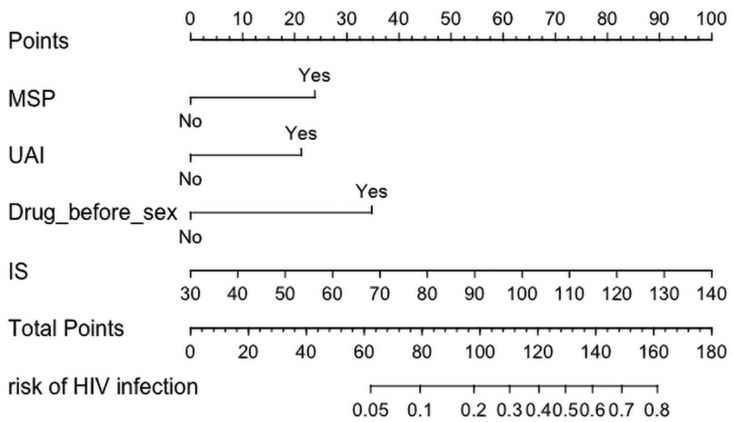
Developed nomogram to assess the HIV infection risk among MSM.

Significant predictors were selected by the LASSO method based on a minimum 1 standard error (1-SE) criterion to obtain the most regularized and efficient prediction model (Figure 2a,b). Among the 15 high-dimensional characteristics of MSM, four variables with non-zero coefficients were identified as risk factors closely associated with HIV infection including IS, UAI, MSP, and drug before sex. Those significant factors selected were included in the multivariate logistic regression to establish the prediction model. Logistic regression analysis demonstrated that all included variables were independent risk factors for HIV infection among MSM, and their coefficients were shown in Table 2. Then, the nomogram diagrams corresponding to the predictive model incorporating multidimensional risk factors was developed as a tool to assess the risk of HIV infection (Figure 3). Each selected risk factor was assigned a corresponding score according to its value on the nomogram. For example, suppose a man had unprotected sexual behavior with multiple sexual partners and took drugs before having sex. With an IS score equal to 32, the total nomogram point for the man was 110. As the nomogram showed, the estimated HIV infection risk of the man was approximately 0.3.

### 3.3. Internal Validation of Prediction Model

**Figure 4:**
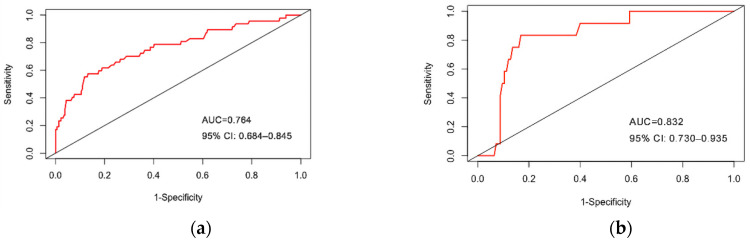
Receiver operating characteristic (ROC) validation of the HIV infection risk nomogram prediction in training set and testing set: (**a**) The area under the receiver operating characteristic curve (AUC) represents the discrimination performance of the model in the training set; (**b**) The area under the receiver operating characteristic curve (AUC) represents the discrimination performance of the model in the testing set.

**Figure 5:**
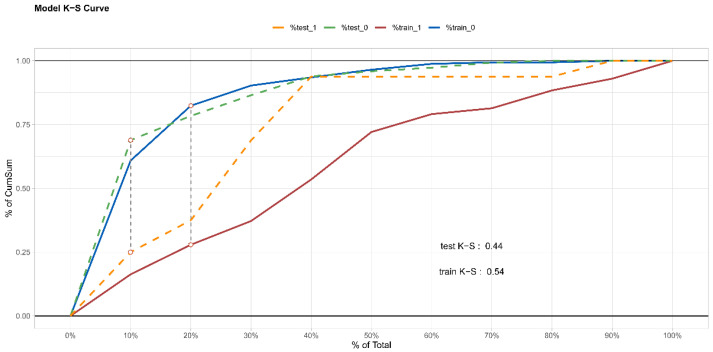
Kolmogorov–Smirnov test curve of the HIV risk prediction model in the training set and in the testing set. The horizontal coordinate of the curve is the “threshold” (the overall sample is divided into 10 equal parts in probability order), and the vertical coordinate is the value of TPR (true positive rate) or FPR (false positive rate), ranging from 0 to 1. The red and blue solid lines indicate the HIV-positive and negative cases in the training set, respectively. The maximum vertical distance between these two curves (gray dashed line) is the KS test value, and the corresponding horizontal coordinate is the threshold value that classifies the model best. The orange and green dashed lines indicate the HIV-positive and negative cases in the testing set, respectively.

**Figure 6:**
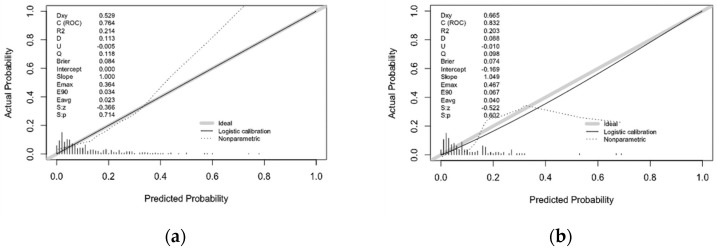
Calibration curves for the prediction of HIV infection risk nomogram The *y*-axis represented the actual risk of HIV infection. The *x*-axis represented the predicted risk of HIV infection. The diagonal dotted line represents a perfect prediction by an ideal model, the solid line represents the performance of the training set (**a**) and testing set (**b**), with the results indicating that a closer fit to the diagonal dotted line represents a better prediction.

**Figure 7:**
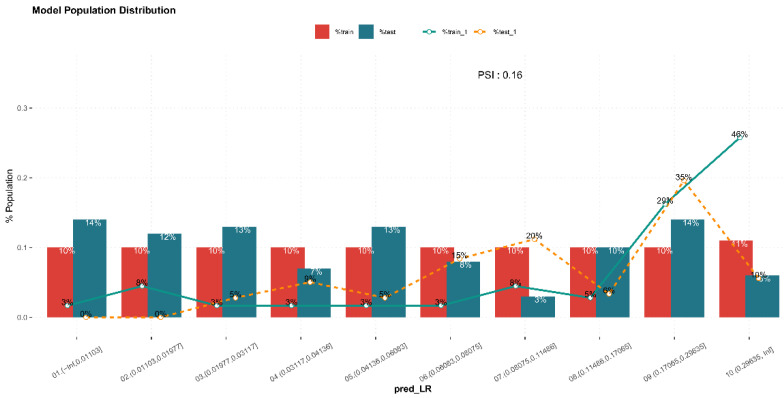
Population distribution plot of HIV infection risk prediction model in the training and testing sets. The horizontal coordinate indicates the 10 binning interval of the general distribution, and the vertical coordinate indicates the percentage of the population. The red and dark green squares indicate the actual population distribution in each binning interval for the training and testing sets, respectively. The green solid line and the orange dashed line indicate the expected population distribution in each binning interval for the training and testing sets, respectively.

The ROC of the prediction model is presented in Figure 4a,b. In the training set, the AUC value was 0.764 (95% CI 0.684–0.845), which indicated that about 76% of the individual HIV infection risk would be correctly predicted by the model. In addition, the K-S value of 0.54 also indicated that the established prediction model has a strong risk differentiation ability (Figure 5). In the testing set, the AUC-ROC was 0.832 (95%CI 0.730–0.935) and the K-S value was 0.44. Therefore, the prediction model has moderately high discrimination for predicting the HIV infection risk among MSM. The calibration curve indicated good agreement between the actual HIV infection probabilities and predicted probabilities made using nomogram in both the training set and testing set (Figure 6a,b). From the Hosmer–Lemeshow test, the predicted and actual probability were highly consistent (training set, *p* = 0.474; testing set, *p* = 0.252). The PSI of 0.16 revealed that the model was stable due to the relatively smaller difference between the distributions of the training and testing sets (Figure 7). To summarize the internal random splitting validation results above, this model has a good predictive capability.

## 4. Discussion

Our study identified four risk factors of HIV infection in MSM from 15 sociodemographic, behavioral, and psychosocial predictors based on LASSO regression, including UAI, MSP, IS, and drug use before sex. We constructed a predictive model that incorporated the above risk factors of HIV infection in MSM, and further developed and validated a HIV infection risk nomogram. The nomogram we developed targeted at MSM has moderate predictive discrimination and calibration in the training set. In addition, the lower PSI also confirmed the relatively good stability of the prediction model. Finally, the internal validation of the nomogram supported its good predictive performance and accuracy in predicting HIV infection risk among MSM.

### 4.1. Association of UAI and MSP with HIV Infection among MSM

UAI and MSP were well-known as the main routes of HIV infection in MSM [33,34]. From adolescents to older men, UAI remained common and put MSM of all ages at higher risk for acquiring and infecting HIV [35,36,37]. In addition to UAI, the mean number of total sexual partners in MSM had increased significantly in recent years, and MSP was emerging as a high-risk factor for HIV infection [38]. Some studies provided further evidence that unprotected sexual behaviors with multiple sexual partners expedited HIV transmission among HIV-positive MSM [39,40]. Our study also confirmed that UAI and MSP were both independent predictors for HIV infection among MSM. However, the above two predictors were not the main HIV infection contributors in this nomogram, which may be related to the duration of the survey regarding previous sexual behavior. It is difficult to assess the impact of high-risk sexual behavior on HIV infection during the lifetime of MSM in cross-sectional studies; therefore, most cross-sectional studies generally investigate short-term (usually the past six months) high-risk sexual behavior among MSM.

### 4.2. Association of Drug Use before Sex with HIV Infection among MSM

Our study found that drug use before sex was a significant predictor for HIV infection among MSM. Previous studies have also reported that drug use was associated with high HIV infection risk [41,42]. This relationship is postulated to be attributed to the acute effects of drug use that can enhance sexual desire and increase intoxicating highs, thus stimulating risk-taking sexual behaviors, such as UAI, MSP [43,44]. In addition to illegal drugs, some popular recreational drugs that but not defined as illegal, such as rush poppers and erectile dysfunction medications, have similarly been confirmed to play a role in increasing rates of sexual risk behavior and HIV infection [45,46,47,48]. Furthermore, several epidemiological studies have also shown a positive dose–response relationship between frequency and number of drugs used or polydrug use and HIV risk to support our findings [49,50,51].

### 4.3. Association of IS with HIV Infection among MSM

Interestingly, our study found the risky role of involuntary subordination in HIV infection among MSM. As far as we know, we were the first to reveal this direct association between involuntary subordination and HIV infection risk among Chinese MSM. We confirmed that the higher the level of IS, the greater the risk of HIV infection. This may be explained by the fact that MSM suffered more discrimination, isolation, and stress than heterosexuals, thus making MSM more prone to feel stuck and low perception (poor social comparison), give up resistance, and abandon themselves when confronted with adversity, eventually resulting in risky sexual behavior [10] and mental disorder such as anxiety and depression [8]. Recently, some studies also confirmed that involuntary subordination fully mediated the relationship between self-esteem and depression among MSM [52,53]. In addition, IS was found to be associated with neediness [9] and dependency [54], which was also compatible with the characteristics of sexual minorities compared to heterosexuals. Currently, the concept of IS was expanded into a range of interconnected feelings and perceptions, including submissive behaviors, entrapment, social defeat and poor social comparison, which may stimulate multiple risk pathways that synergistically increase the risk of HIV infection, thus partly explaining the higher contribution of IS in the nomogram. This finding could inform future research, especially when additional mental health variables are available for predictive purposes. More research is needed to focus on the potential impact of involuntary subordination on HIV infection and provide further insights into the unclear causal relationship between IS and HIV infection.

### 4.4. Limitation

There were several limitations to this study. Firstly, since this study was a cross-sectional study, the ability to infer causality from these findings was insufficient. Secondly, participants’ responses might not be completely honest because sexual-related questions are relatively private in China and the questionnaire in our study was completed through face-to-face interviews. Therefore, it was inevitable to generate biases from the self-reported data collected. Finally, given the difficulty of including all psychosocial and behavioral factors that may be associated with HIV infection in the model, large prospective cohort studies containing more comprehensive variables are needed in the future to further expand the applicability of this HIV infection risk assessment tool.

## 5. Conclusions

Our study found that MSP, UAI, IS, and drugs before sex were significant predictors in terms of identifying risk for HIV infection. Furthermore, we developed and externally validated an HIV infection risk assessment model especially incorporating psychosocial and behavioral factors for MSM in China. The risk nomogram had a relatively high performance and stability in predicting HIV infection risk among MSM.

## Figures and Tables

**Table 1:** Demographic, psychosocial and behavioral characteristics of the 547 MSM enrolled in the study according to randomization to the training and testing sets.

Characteristic	Total Populations (*n* = 547)	Training Set (*n* = 410, 75%)	Testing Set (*n* = 137, 25%)	*p*-Value
Age (years old)	28.0 (25.0, 33.0)	28.0 (25.0, 33.0)	27.0 (24.5, 34.0)	0.933
Employment status				0.991
Employed	447 (81.7)	335 (81.7)	112 (81.8)	
Unemployed	100 (18.3)	75 (18.3)	25 (18.2)	
Highest education level				0.858
Senior high school or less	157 (28.7)	119 (29.0)	38 (27.7)	
College degree or above	390 (71.3)	291 (71.0)	99 (72.3)	
Current marital status				0.195
Single	434 (79.3)	329 (80.2)	105 (76.6)	
Married ^1^	82 (15.0)	62 (15.1)	20 (14.6)	
Divorced or widowed	31 (5.7)	19 (4.7)	12 (8.8)	
Income(CNY)				0.332
<3000 ^2^	133 (24.3)	99(24.1)	34(24.8)	
3000–6000	211 (38.6)	152(37.1)	59(43.1)	
>6000	203 (37.1)	159(38.8)	44(32.1)	
Residence status				0.079
Local	389 (71.1)	127 (31.0)	31 (22.6)	
Non-local	158 (28.9)	283 (69.0)	106 (77.4)	
Sexual orientation				0.714
Non-homosexual	157 (28.7)	116 (28.3)	41 (29.9)	
Gay/homosexual	390 (71.3)	294 (71.7)	96 (70.1)	
Have had a VCT				0.822
No	251 (45.9)	187 (45.6)	64 (46.7)	
Yes	296 (54.1)	223 (54.4)	73 (53.3)	
Alcohol use before having sex				0.415
No	278 (50.8)	213 (52.0)	65 (47.4)	
Yes	269 (49.2)	197 (48.0)	72 (52.6)	
Drug use before having sex				1.000
No	530 (96.9)	397 (96.8)	133 (97.1)	
Yes	17 (3.1)	13 (3.2)	4 (2.9)	
MSP				0.502
No	250 (45.7)	184 (44.9)	66 (48.2)	
Yes	297 (54.3)	226 (55.1)	71 (51.8)	
UAI				0.188
No	249 (45.5)	180 (43.9)	69 (50.4)	
Yes	298 (54.5)	230 (56.1)	68 (49.6)	
Involuntary subordination	80.52 ± 18.06	80.31 ± 18.23	81.12 ± 17.57	0.649
Social support	61.0 (51.0, 70.0)	62.00 (51.0, 70.0)	59.00 (50.5, 69.0)	0.448
Sexual compulsivity	23.0 (19.0, 26.0)	23.00(19.0, 26.0)	23.00(20.0, 26.0)	0.906

^1^ Marital status refers to only heterosexual marriage. Homosexual marriage is still not legalized in Mainland China. ^2^ CNY 3000 equivalent to USD 450; 6000 equivalents to USD 900. Note: VCT, voluntary HIV counseling and testing; MSP, multiple sexual partners; UAI, unprotected anal intercourse. Data are mean ± SD, median (interquartile range) or *n* (%).

**Table 2:** Logistic regression analysis of the predictors for the risk of HIV infection among MSM.

Intercept and Variables	Estimate	Prediction Model		
		Wald Values	Odds Ratio (95% CI)	*p*-Value
Intercept	−6.862	51.835	0.000 (0.000, 0.006)	<0.001
UAI	0.931	5.698	2.536 (1.219, 5.703)	0.017
MSP	1.043	6.720	2.837 (1.338, 6.591)	0.010
Drug use before sex	1.522	5.323	4.579 (1.184, 16.437)	0.021
Involuntary subordination	0.040	17.161	1.041 (1.022, 1.061)	<0.001

Note: CI, confidence interval.

## Data Availability

The data presented in this study are available on reasonable request from the corresponding author.
